# Supplementary material for: Preservation of neurons in an AD 79 vitrified human brain
Source: PLoS One. 2020 Oct 6;15(10):e0240017. doi: 10.1371/journal.pone.0240017 (PMC7537897; doi:10.1371/journal.pone.0240017)
Supplement: S1 File — (PDF) [file pone.0240017.s001.pdf]

Supporting Information

1 Morphological analysis

To perform the morphological analysis the first step is to define the most likely image contours. The main difficulty in this process is to identify as well as possible the dividing surface between image and background. This means that the recorded images must be treated offline with a specific digital image processing to recover correct information starting from the SEM image containing all the frequencies, noise included. In order to amplify the contours of the objects and to reduce the noise, the images were pre-processed by means of the Wavelet Transform (WT). Once the most likely contours had been defined, the images were cut in order to select only the area used for the neural analysis. A specific tool in Matlab® based on the neural network theory was developed.

1.1 The Wavelet Transform

The WT is able to detect the presence of details usually lost when filters reduce the noise of the signal. The WT gives a time–frequency representation of the signal providing a high resolution in the time and in the frequency domain. Wavelets, like sinusoidal functions in Fourier analysis, are used for representing signals [1]. In fact, the WT is a complete orthonormal system (see the Parseval's theorem [2]). It is easy to show that W's form an orthonormal basis [2-3]. It can been show that the ‘‘detail space” W_j is itself made up of three orthogonal subspaces. Such three sets of wavelets correspond to specific spatial orientations [4]: the horizontal direction, the vertical direction and the diagonal. For more detail, see [2,5-6]. Here, it is important to enhance that the space, where the wavelets are set, is a multidimensional Hilbert space, *i.e.*, a complete pre-Hilbert space. In this application, the R^n space satisfies the properties of the Hilbert space, an orthonormal complete system (basis), that allows the study of images with an accuracy and resolution that cannot be achieved by other methods.

The WT is mathematically defined as a convolution between the two-dimensional signal (*i.e.*, image) and the chosen wavelet function. This function has the characteristic to dilate and contract. Its contracted version detects the high-frequency components in the original signal while, the dilated versions, detects the low-frequency components. In this approach, the WT is used to decode the two-

dimensional image obtained by the SEM. Furthermore, a suitable algorithm allows to decompose the two-dimensional signal (matrix image). In this way it is possible to study the signal (*i.e.,* image matrix) along three different directions: horizontal, vertical and diagonal. The image is then discretized along a one-dimensional scale and, consequently, an algorithm based on the fuzzy neural network [7,8] can be applied. Such a methodology is very suitable when the systems show non-linear characteristics (*e.g.*, singularity or discontinuity).

In the mathematical field, a singularity is a point where a function is not differentiable although it is differentiable around it. The singularities, also called discontinuities, are defined as sudden changes of a signal that occur in very short time. Such singularities are often suppressed by the use of high-cut filter. The identification of discontinuities, in signals and images as well as their localization do not depend only on the filtering process as it might appear at first sight. The singularities into signals represent the trend of physical quantities observed [9]. They have a high content of information and denote the occurrence of transient phenomena and rare events. In a 2D signal (*i.e.*, image) the singularities represent the contours of objects, their changes in the properties of absorption or reflection of the bodies, lighting variations, temperature variations, important thermal gradients, etc. Crests of roughness may be thought as a smoother version of singularities where large signal changes occur over slightly broader time changes. A theory for examining the singularities of functions using the Wavelet Transform was developed [9,11], which has been applied here to identify and characterize the crests that make up the surface roughness of an object inside the image.

1.2 The Self-Learning Neural Network

As reported in the Introduction, a Self-Learning Neural Network (SLNN) was developed in order to operate both a denoising and an enhancement of features showed by the images detected and amplified by the WT. The SLNN was based on three layers (Input, Hidden and Output). In each of them there will be $M \times N$ neurons (assuming an $M \times N$ image). Neurons belonging to the same level have no connection among them. Each neuron corresponds to one pixel. Each neuron in a layer is connected to the corresponding neuron in the previous layer and its neighboring neurons. Note that the nodes of two consecutive layers are linked by a weighted vector.

Each node was activated both in accordance with the inputs received from the total of nodes belonging to the preceding layer and from the activation function of the same node. The total of input to the *i*-th node for each layer is:

$$I_i = \sum_j w_{ij} o_j \qquad (1)$$

where o_j is the output of the *j*-th node of the preceding layer and w_{ij} is the weighted link between the *i*-th node of a layer and the *j*-th node of preceding layer.

The output of the *i*-th node is:

$$o_i = f(I_i) \qquad (2)$$

where $f(\cdot)$ is the sigmoidal activation function expressed as:

$$f(\cdot) = \frac{1}{\sqrt{2\pi}} e^{\frac{\bar{X}-x}{2\pi\sigma}} \qquad (3)$$

where $\bar{X}$and σ were the mean and the standard deviation respectively of the values of the nodes selected by the neighborhood system. Here, we used an array composed of five nodes (figure 1) [9-10].

5	4	3	4	5
4	2	1	2	4
3	1	(i, j)	1	3
4	2	1	2	4
5	4	3	4	5

Figure 1. Neighborhood system

The input layer takes as input the image generated by the WT, as described in the previous paragraph, and each neuron of the layer uses (3) as transfer function. The output of the 1st layer (but this is valid for all the three layers composing the neural network) was passed to the 2nd layer using the (1). When the net is analyzing the *i*-th point, we took as neighborhood the points preceding *i* and the two points following *i*. This passage was done for each layer. The output of the 3rd layer was

then compared with the target signal (*i.e.*, the original image) obtaining the error signal which was passed to the 1st layer in order to recalculate the weights w_{ij}.

The input of a neuron of the input layer is given by a real number in [0, 1] proportional to the gray level of the corresponding pixel of the current analyzed image. Since we are interested in eliminating noise and extracting compact regions spatially, all the initial weights must be equal to 1. No external bias will be imposed to the weights. A random initialization of weights could result in a loss of the regions extracted in a compact manner as such an initialization could induce a pseudo noise image. Hence, we underline why the weights, at the first epoch, were set equal to 1.

The formula, named square variation index, implemented to recalculate the weights was chosen in order to minimize the error between the signal analyzed by net and the signal to which the net tries to fit the input signal:

$$\Delta w_{ji} = \{\beta(1 - o_j)f'(I_j)o_i\}. \qquad (4)$$

At the end of each iteration the output signal, obtained from the 3rd layer, was plotted in order to appreciate the improvement performed by the neural application. An example of the 7-th iteration from the 1st up to the 3rd layer is showed in the figures 2, 3 and 4 below. Figure 5 shows the target image.

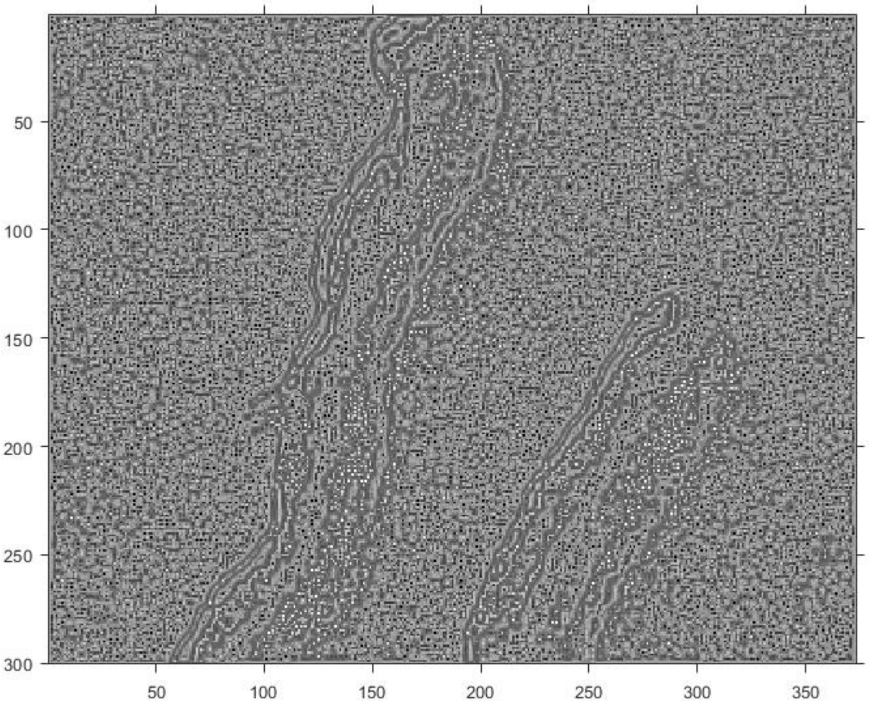

Figure 2. Epoch 7 – 1st Layer – 7th Iteration

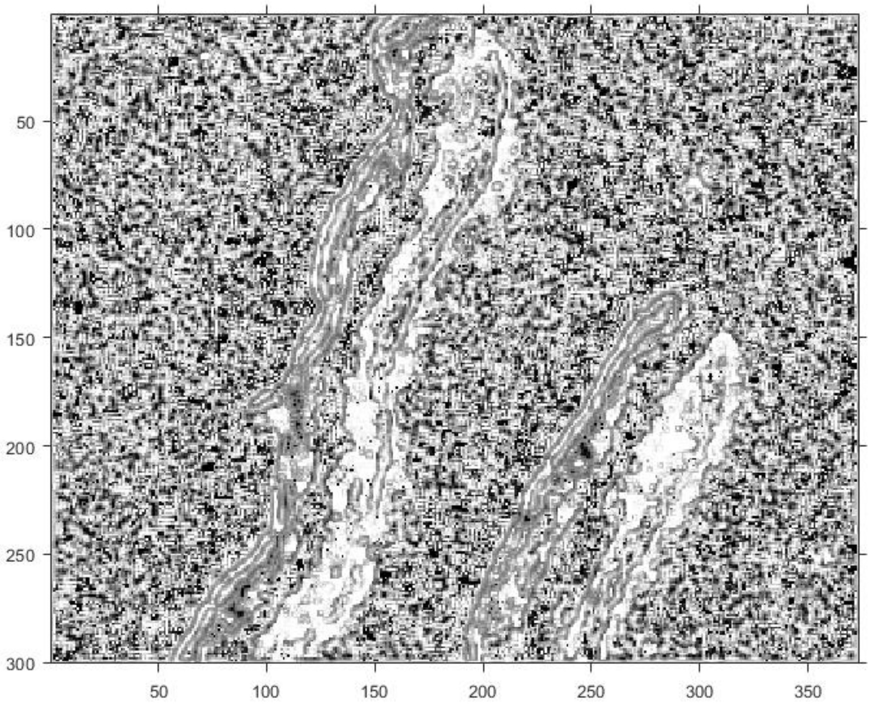

Figure 3. Epoch 7 – 2nd Layer – 7th Iteration

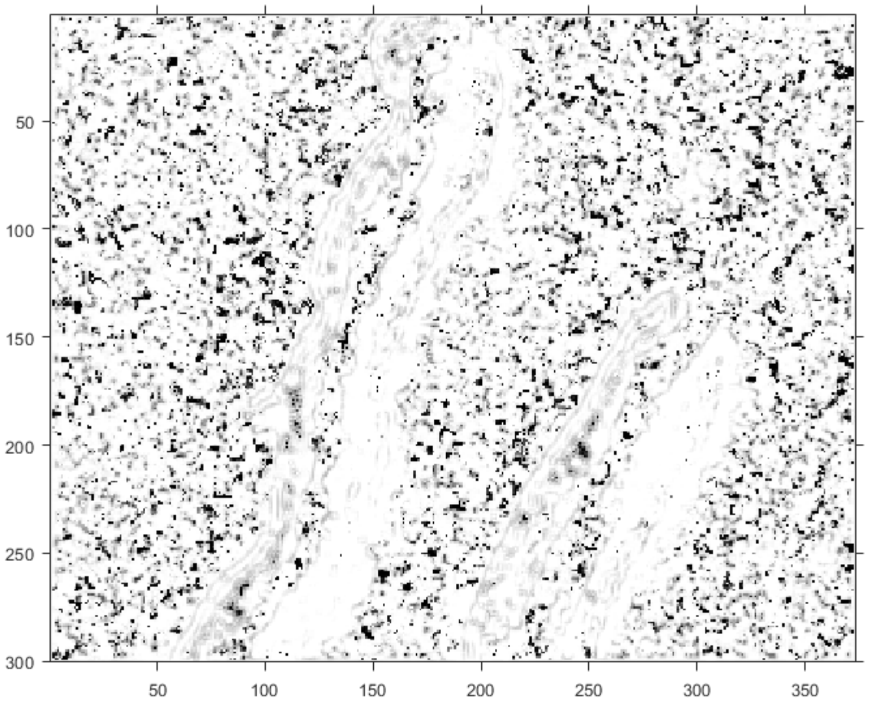

Figure 4. Epoch 7 – 3rd Layer – 7th Iteration

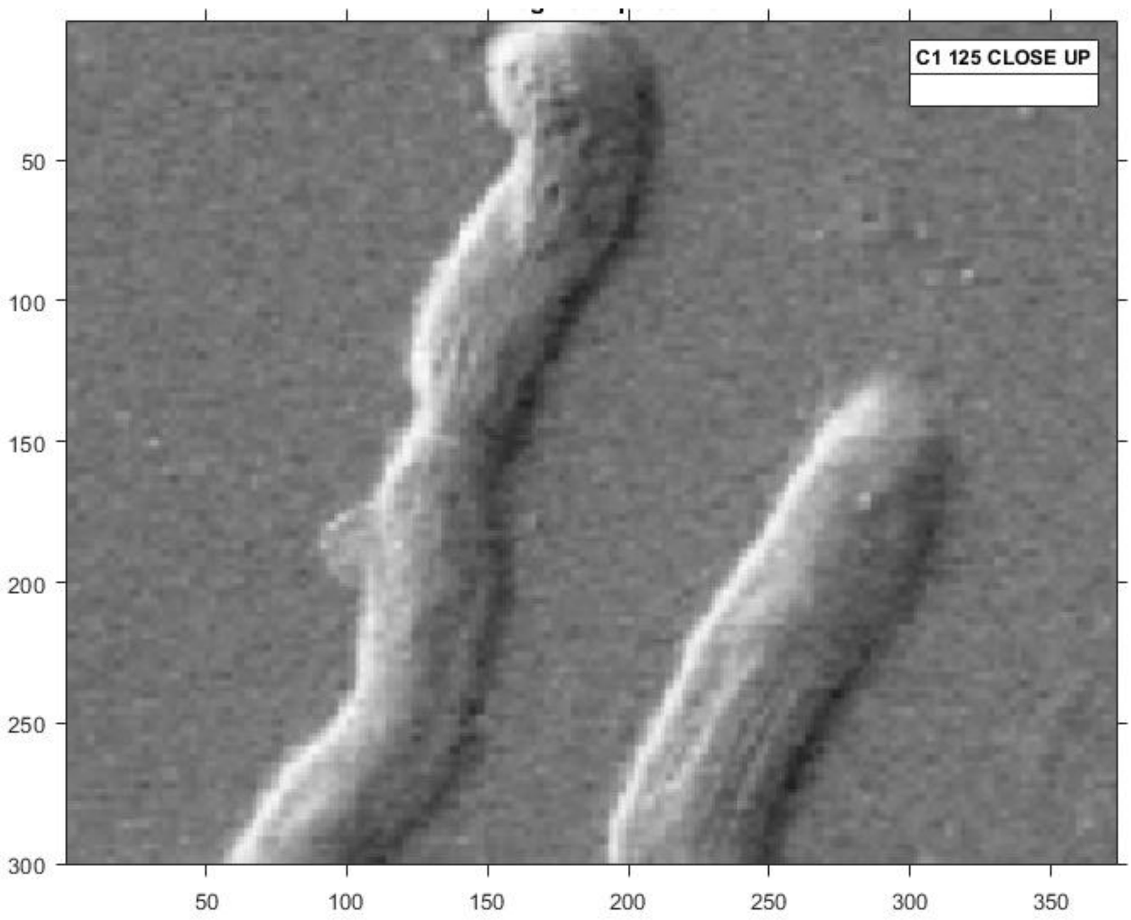

Figure 5. Target image (SEM image)

2 Morphometric image processing

The pre-processed morphological images present information that does not allow to extract directly the outlines of interest in a clear and precise form. The morphometric image processing is essential to identify objects in the image and making reliable measurements. To overcome this obstacle, a processing procedure divided into three fundamental phases has been developed in Matlab®:

i. Image optimization: to highlight the contours of the ROI;
ii. Research of contours: within a mask created around the ROI, the contours that best approach the object itself with a segmentation procedure of the foreground and background image using the algorithm of "active contours", and getting the ROI related to the object;
ii. Measurement: evaluation geometric parameters relating to the ROI.

2.1 Processing Procedure

Below is a brief description of the steps of the processing procedure. The optimization phase aims to modify the starting image to enhance the contours of the objects in it. In detail, this process is obtained by subtracting the most recurrent gray intensity value in the image and by improving the contrast through adaptive equalization of the gray levels carried out with the use of the image histogram, [12]. The results of this operation are shown in figure 6. The contours present in the image are evident (right), *i.e.*, the boundary lines between different gray-level areas. Such contours are not evidenced in the original SEM image (left). The enhancement of these boundary lines simplifies the next phase in which the most likely contour of the object is sought.

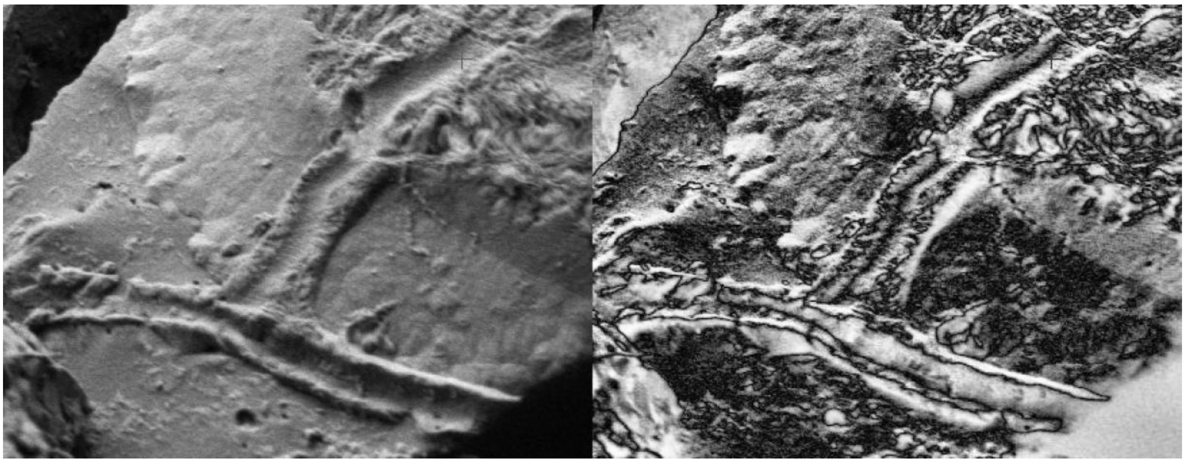

Figure 6. Image optimization: on the left the original SEM image, on the right the enhanced contours

Second stage of processing: the outline of the object of interest is identified. First of all, the ROI is by tracing a polygonal around the object to be analyzed. The mask thus obtained represents the trigger point of an iterative segmentation procedure that moves the contours of the region of interest until they match them with the closest ones present in the image through the "active contours" algorithm [13-15]. The result of this step is shown in figure 7 where it is possible to observe the shape of the identified object (highlighted in blue).

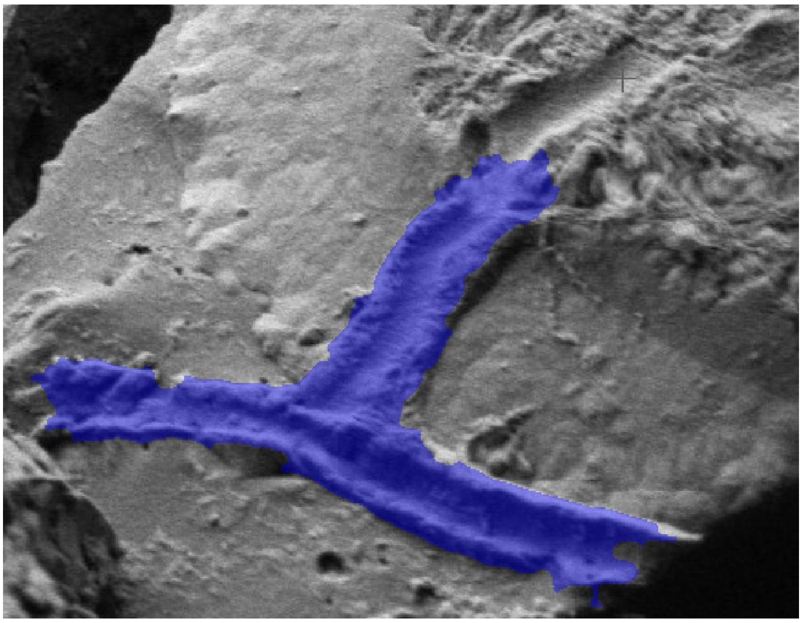

Figure 7. Shape of the identified object

The last step of the morphometric image processing performs the measurements (*i.e.*, the area, the length of the "skeleton", the perimeter of the object and the average thickness) of the identified shape, in the hypothesis that the object has a shape with a dominant dimension. Figure 8 shows the identification of the most likely perimeter and skeleton extracted from the ROI.

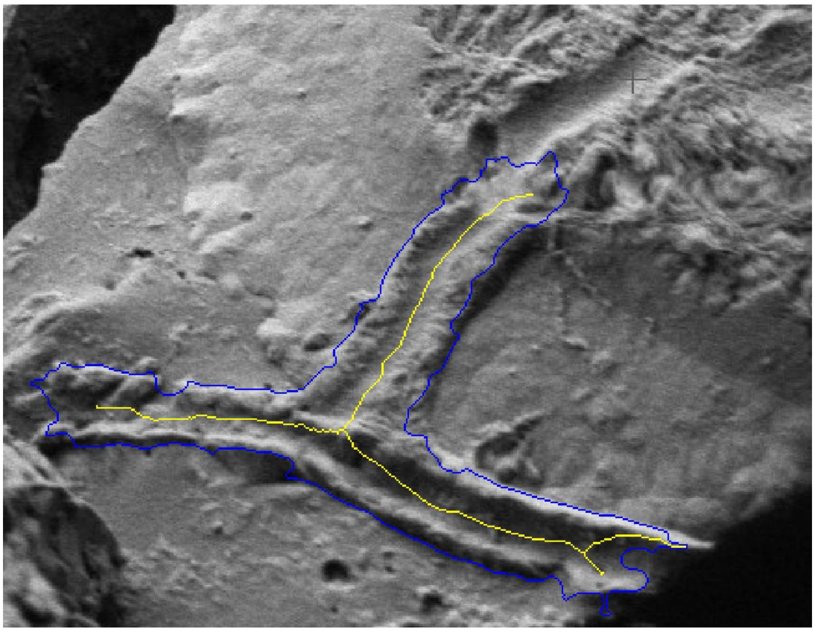

Figure 8. Perimeter (in blue) and skeleton (in yellow) of the ROI

2.2 Measurements

In Table 1, the measurements performed on the ROI are showed.

Area ($pixel^2$)	Perimeter (pixel)	Length of the skeleton (pixel)
22924	1237,108	547,216

Table 1. Main measurements of ROI

References

1. Kaiser G. A Friendly Guide to Wavelets. Birkhäuser, 1999.

2. Daubechies I. Ten lectures on Wavelets. Ed. SIAM, Philadelphia, Pennsylvania, 1992.

3. Härdle W, Kerkyacharian G, Picard D, Tsybakov A. Wavelets Approximation and Statistical Applications. Springer, Berlin, 1998.

4. Mallat SG. A theory for multiresolution signal decomposition: the wavelet representation. IEEE Trans. Pattern Anal. Mach. Intell. 1989; 11:674–693.

5. Horn BKP. Understanding image intensities. Artificial Intell. 1977.

6. Ogden RT. Essential Wavelets for Statistical Applications and Data Analysis.

Birkhäuser, Boston, 1997.

7. Zadeh LA. Fuzzy sets. Information and Control. 1965; 8(3):338-353.

8. Chiu S. Fuzzy model identification based on cluster estimation. Journal of Intelligent and Fuzzy Systems, Vol.3, 1994.

9. Mallat S, Hwang WL. Singularity detection and processing with wavelets, IEEE Trans. Inform. Theory. 1992; 38(2):617–643.

10. Krishnapuram R, Frigui H, Nasraoui O. Fuzzy and possibilistic shell clustering algorithms and their application to boundary detection and surface approximation – Part I. IEEE Transactions on Fuzzy Systems. 1995; 3(1):44-60.

11. Niola V, Nasti G, Quaremba G. A problem of emphasizing features of a surface roughness by means the Discrete Wavelet Transform. J Materials Process Technol. 2005; 164:1410-1415.

12. Zuiderveld K. Contrast Limited Adaptive Histograph Equalization. Graphic Gems IV. San Diego, Academic Press Professional. 1994; pp. 474–485.

13. Chan TF, Vese LA. Active contours without edges. IEEE Transactions on Image Processing. 2001; 10(2):266-277.

14. Caselles V, Kimmel R, Sapiro G. Geodesic active contours. Int J Computer Vision. 1997; 22(1):61-79.

15. Whitaker RT. A level-set approach to 3d reconstruction from range data. Int J Computer Vision.1998; 29(3):203-231.
