# Supplementary material for: Preservation of neurons in an AD 79 vitrified human brain
Source: PLoS One. 2020 Oct 6;15(10):e0240017. doi: 10.1371/journal.pone.0240017 (PMC7537897; doi:10.1371/journal.pone.0240017)
Supplement: Attachment — Submitted filename: Respons to Reviewers.docx [file pone.0240017.s002.docx]

**Rebuttal letter – Ref.: PONE-D-20-24415**

Title: Preservation of Neurons in an AD 79 Vitrified Human Brain

**Response to the comments from the editor and reviewers**

We wholeheartedly agree with comments from the editor and reviewers therefore we made a revision of the critical issues. In particular, following the suggestions of reviewer #2, we have treated in more depth and clarified the neuronal origin of the structure shown in figure 4, in order to avoid any possible misinterpretation concerning non-myelinated axons vs small blood vessels.

As also suggested, we ensured to refer to Tables 1 & 2 in the text, and a caption for all of the figures has been added at the end of the manuscript. The four additional citations have also been added and renumbered within the text and bibliography as well. We have also improved the description of the databases used for the expression study, both in “Results” and “Materials and Methods” sections.

***Reviewer #1****: This is an excellent analysis of the preservation of neural tissue by vitrification from a young male from Herculano. Authors provides detailed characterization of neurons and axons using SEM analysis.*

We thank the reviewer for his/her positive and insightful comments on the manuscript.

***Reviewer #2****: This contribution is probably the most surprising article I have had the opportunity to review. Indeed, it is a difficult task to provide the authors with a well-argued opinion. Having a chance of inspecting a 2000-year-old vitrified CNS specimen of course is a very rare event. Here the authors took advantage of natural vitrification process enabling ? for the first time if I am right- the accurate identification of neuronal structures at an unprecedented level of resolution. Taken together, this can be viewed as a "neuroarcheological" report.*

*My only concern is related to the method conducted for identifying neuronal processes from any other CNS structure like small blood vessels. As indicated in page 5, it seems that the main criteria is that identified tubular structures are smaller in diameter that blood vessels in the CNS. Although this is overall fine to me, I am wondering to what extent this will suffice. For instance, the structure shown in Figure 4 is here taken as a good example of non-myelinated axon arising from a spinal cord neuron. Although I am obviously not too much familiar with the conducted analytical procedure, I guess this could also be considered as a blood vessel of small caliber.*

*In summary, I guess this undoubtedly is a very rare example of neuroarcheology taken from a very rare event and of course of great interest from potential readers, likely representing a wide audience. Nevertheless and bearing in mind my background in neuromorphology, I guess the authors should make a greater effort in better explaining the criteria used for ensuring the neuronal origin of some of the observed structures.*

We thank the reviewer for his/her suggestion. We have now included two references (Müller et al., 2008; Schmid et al., 2019) in the main text showing that the smallest capillaries in the human brain have diameters ranging from 8 to 1 µm and a wall thickness of about 1 µm (Figure 1).

**Figure 1** . The typical diameters/wall thicknesses of blood vessel for humans and mice are given in micrometers. The smallest capillaries have diameters of a very few micrometers and a wall thickness of about one micrometer (from Müller et al., 2008).

In Figure 4 of our manuscript the mean diameter of a non-myelinated axon is 452·50 ∓ 20·50 nm, that is significantly inferior to the minimal dimension of the diameter of a capillary as reported in literature. Moreover, we can add two other important morphological details that can confirm our description. The first one is that this structure originates in fact from the changing curvature of the cellular membrane and it extends for several microns away from the cell body. To be clearer, here below we show a comparison between our SEM image (left) and a TEM image (right) of the axon initial segment (red arrows) of a cerebral cortical pyramidal neuron (Bender et al, 2012) that shows the same section that we found in our sample. Starting with this description we can assume that in our sample we have found a section of axon initial segment that is a non-myelinated axon (Palay et al., 1968). The last point that validates our description is that the wall of this structure is around 70 nm, that is far below the size of the wall thickness of the capillary ranging around 1 / 0.5 µm (Müller et al., 2008).

Therefore, we have added the following sentences (page 6, lines 166-175 of Revised Manuscript with Track Changes):

"… diameter (nm, mean ∓ SD) (452·50 ∓ 20·50) (Fig 4A). Several evidence seems to confirm the neuronal origin of such structure: i. its diameter is significantly inferior to the minimal diameter of a capillary, the smallest one of which in the human brain have diameters ranging from 8 to 1 µm, and a wall thickness of about 1 µm [38,39]; ii. it originates from the cellular membrane and it extends for several microns away from the cell body; iii. its high similarity with the axon initial segment of a cerebral cortical pyramidal neuron [40]; iiii. the thickness of its wall (ca 70 nm) is far below the size of the wall thickness of a capillary (ca 1 to 0.5 µm) [38]. On this basis, we can assume that in our sample we have found a section of an axon initial segment which is a non-myelinated axon [41]".

Additional Bibliography

38. Müller B, Lang S, Dominietto M, Rudin M, Schulz G, Deyhle H et al. High-resolution tomographic imaging of microvessels. In Developments in X-Ray Tomography VI, Stuart R. Stock (ed), Proc. of SPIE Vol. 7078, 70780B, 2008; 0277-786X/08/$18 · doi: 10.1117/12.794157.

39. Schmid F, Barrett MJ, Jenny P, Weber B. Vascular density and distribution in neocortex. Neuroimage. 2019; 197:792-805.

40. Bender KJ, Trussell LO. The physiology of the axon initial segment. Ann Rev Neurosci. 2012; 35:249-265.

41. Palay SL, Sotelo C, Peters A, Orkand PM. The axon hillock and the initial segment. J Cell Biology. 1968; 38(1):193-201.
